# Oxygen‐insensitive nitroreductase *E. coli* NfsA, but not NfsB, is inhibited by fumarate

**DOI:** 10.1002/prot.26451

**Published:** 2022-12-13

**Authors:** Martin A. Day, David Jarrom, Navina Rajah, Peter F. Searle, Eva I. Hyde, Scott A. White

**Affiliations:** ^1^ School of Biosciences University of Birmingham Birmingham UK; ^2^ Institute for Cancer and Genomic Sciences University of Birmingham Birmingham UK

**Keywords:** flavoprotein, FMN, fumarate, nitrofuran, Nitroreductase, prodrug

## Abstract

*Escherichia coli* NfsA and NfsB are founding members of two flavoprotein families that catalyze the oxygen‐insensitive reduction of nitroaromatics and quinones by NAD(P)H. This reduction is required for the activity of nitrofuran antibiotics and the enzymes have also been proposed for use with nitroaromatic prodrugs in cancer gene therapy and biocatalysis, but the roles of the proteins *in vivo* in bacteria are not known. NfsA is NADPH‐specific whereas NfsB can also use NADH. The crystal structures of *E. coli* NfsA and NfsB and several analogs have been determined previously. In our crystal trials, we unexpectedly observed NfsA bound to fumarate. We here present the X‐ray structure of the *E. coli* NfsA‐fumarate complex and show that fumarate acts as a weak inhibitor of NfsA but not of NfsB. The structural basis of this differential inhibition is conserved in the two protein families and occurs at fumarate concentrations found *in vivo*, so impacting the efficacy of these proteins.

**Abbreviations**
NFZ: nitrofurazone
PEG: polyethylene glycol

## 1 INTRODUCTION

*Escherichia coli* NfsA and NfsB (Nitrofuran sensitive A and B) are the founding members of two families of nitroreductases that occur in a large number of bacteria (reviewed in 1). They were initially discovered as mutations in these genes render *E. coli* resistant to nitrofuran antibiotics. In addition to their importance for nitrofuran antibiotics, the ability of the enzymes, particularly NfsB, to reduce nitroaromatics to cytotoxic hydroxylamines has been used for cancer gene therapy with nitroaromatic prodrugs such as CB1954 (reviewed in 2) and with nitroaromatic probes for hypoxia, in cell ablation studies^3^, ^4^, ^5^ and in bio‐remediation of TNT.^6^, ^7^ These two families of nitroreductases form part of a superfamily of proteins, with over 20 000 sequences known to date, that catalyze a diverse range of reactions^8^, ^9^ and have been proposed for use in biochemical engineering.^9^, ^10^

NfsA and NfsB are FMN‐containing proteins that use NAD(P)H to reduce quinones and nitroaromatics, with a substituted enzyme (ping‐pong) mechanism.^11^, ^12^ They react via 2‐electron steps, without the formation of free radicals, and hence are classified as oxygen‐insensitive, as opposed to oxygen‐sensitive nitroreductases that give 1‐electron radical intermediates.^13^ NfsA uses NADPH preferentially as the cofactor,^11^, ^14^ whereas NfsB can use either NADH or NADPH with similar affinity.^12^, ^15^ They have a similar range of known substrates,^11^ but the *in vivo* substrates and roles of the enzymes are not known. However, despite decades of use of nitrofuran antibiotics, resistance mutants have not spread and mutants show reduced growth rates,^16^ suggesting that the genes are important *in vivo*. Both genes are upregulated by MarA^17^, ^18^ and SoxS,^19^, ^20^ while NfsA is also regulated by OxyR. The regulation by SoxS and OxyR suggests that the proteins have roles in combating oxidative stress, while regulation by MarA indicates roles in reducing the effects of environmental pollutants.

The structure of *E. coli* NfsA has been determined in the absence of substrates by Kobori et al. (1F5V).^21^ Recently, we determined the structure of NfsA from *E. coli* in complex with the substrates nitrofurantoin, quinone, with the product hydroquinone and with an inhibitor bound FMN^22^ and, separately, with the cofactor NADP^+^ ^23^. We and others have also determined the structure of free NfsB^24^, ^25^ and of NfsB in complex with nicotinic acid^26^ and nitrofurazone.^27^ Several structures of NfsA and NfsB homologs^28^, ^29^ and mutants have also been determined. In our initial attempts to crystallize NfsA, we unexpectedly found a four‐carbon, dicarboxylic acid in the active site. In this work, we show the structure of the NfsA complex with fumarate and kinetic inhibition assays with both NfsA and NfsB. NfsA but not NfsB is inhibited at *in vivo* concentrations of fumarate, but only much higher concentrations of succinate. This inhibition could be significant for future use in biosynthesis and therapeutic applications and may help indicate the *in vivo* role of NfsA.

## 2 MATERIALS AND METHODS

### 2.1 Protein expression and purification

*E. coli* NfsA was overexpressed in *E. coli* BL21 (λDE3) without any tags, from the pET‐24 derivative pPS1341A1, encoding NfsA under the control of a T7 promoter, as described in Vas et al.^14^ It was purified as described previously, using ammonium sulfate precipitation, hydrophobic interaction chromatography on phenyl sepharose, ion exchange chromatography on Q sepharose, followed by size exclusion chromatography on Sephacryl 200 or Superdex 75. *E. coli* NfsB was over expressed from a pET‐11c plasmid derivative and purified using similar methods.^26^

Protein concentrations were estimated by Bradford assay^30^ or by determining the absorbance at 280 nm where both the protein and the cofactor absorb, and correcting for excess FMN by measuring the absorbance at 454 nm, where only FMN absorbs. The molar absorbances used were 12 200 M^−1^ cm^−1^ for FMN at 454 nm, 20 970 M^−1^ cm^−1^ for FMN at 280 nm, 31 190 M^−1^ cm^−1^ for NfsA at 280 nm, and 22 460 M^−1^ cm^−1^ for NfsB, based on their amino acid composition.^31^

### 2.2 X‐ray crystallography

Crystals were grown by a sitting‐drop method. Purified NfsA was concentrated to between 10–16 mg/ml, and then dialyzed into 100 mM imidazole, pH 7.0. The mother liquor for crystallization contained 100 mM imidazole pH 7 as a buffer and 27% PEG 3000 (Fluka Analytical, St. Gallen, Switzerland) as a precipitant, in the presence of 30 mM fumarate. Crystals appeared within 24 h and generally reached full size within 48 h. To cryo‐protect the crystals, they were soaked in mother liquor containing increasing concentrations of ethylene glycol, lowering the concentration of the PEG precipitant alongside each incremental increase in cryo‐protectant. The crystals were then flash cooled in liquid nitrogen.

Data were collected on a Rigaku 007HF generator with a Saturn CCD detector mounted on a 4‐circle kappa goniometer.

Diffraction images were indexed, integrated, and processed using MOSFLM,^32^ iMOSFLM,^33^ or XDS.^34^ Datasets were combined and scaled using POINTLESS and SCALA^35^ and data quality was assessed using XTRIAGE.^36^ All structures were solved by molecular replacement with PHASER,^37^ using the published NfsA structure PDB entry 1F5V for NfsA^21^ as the starting model. Structures were refined using REFMAC5^38^ and then with PDB‐Redo.^39^ Models were built and modified using Coot.^40^ Final models were validated using MOLPROBITY^41^ and POLYGON.^42^ The structural figures were drawn using UCSF Chimera 1.13.1.^43^

### 2.3 Steady state enzyme assays

Steady‐state kinetic assays were monitored spectrophotometrically, over 1–2 min, as described previously^27^ Experiments were performed in 10 mM Tris–HCl pH 7.0, at 25°C, at 50 mM total ionic strength for fumarate, assuming that is doubly charged at this pH. Succinate showed weak inhibition so the reactions with NfsA were done at higher concentrations of succinate, which required maintaining a higher ionic strength, 150 mM. Reactions were initiated by the addition of a small quantity of enzyme (~10 nM). Nitrofurazone was dissolved in 90% DMSO, 10 mM Tris–HCl pH 7.0, and kinetic experiments included a final concentration of 4.5% DMSO.

Reactions were measured at 420 nm using molar absorbance change of 4 300 M^−1^ cm^−1^ for the reaction.

Initial reaction rates, *v*_i_, were measured for a range of concentrations of one substrate in the presence of a fixed concentration of the other substrate, with and without the inhibitor (*I*) at a series of inhibitor concentrations. All data was analyzed using Sigmaplot 14.5 with equal weighting of points. The type of inhibition, and the inhibition constants were initially evaluated by fitting the data for each substrate separately to the Michaelis‐Menten equation to obtain values for the apparent *k*_cat_, apparent *K*_m_, and inhibition constants. For NfsA, the global *k*_cat_, *K*_m_, and inhibition constants *K*_i_ were then determined by fitting all data for both substrates to Equation 1, describing inhibition of both halves of the ping‐pong reaction, or for inhibition of only one half of the reaction; that is, competition with only substrate A or only substrate B.

(1) viE=kcatABKmAB1+IKiA+KmBA1+IKiB+AB

where (*A*) is the concentration of Nitrofurazone, (*B*) is the concentration of NADPH and (*I*) is the concentration of inhibitor.

## 3 RESULTS AND DISCUSSION

### 3.1 Crystallography

*E. coli* NfsA was purified from an over‐expressing strain of *E.coli*, as described previously^14^ and crystallized in the absence of ligands. Unexpectedly, our initial crystals of NfsA showed a four‐carbon, dicarboxylic acid bound in the active site; the resolution was insufficient to distinguish whether this was the saturated succinate (butanedioate), or fumarate which contains a central, trans, double bond ([E] but‐2‐ene dioate). Addition of succinate or fumarate to the crystallization solution improved the quality of the crystals. The ligand was not seen in the structure when a different source of PEG was used to crystallize the protein, and so is likely to have come from impurities in the PEG. Crystallization of the protein in the new PEG in the presence of fumarate allowed us to confirm the structure and interactions of the acid and the protein (Table 1).

**TABLE 1:** X‐ray crystal data collection and refinement statistics for NfsA bound to fumarate.

PDB code	
Data collection	8AJX
Wavelength (Å)	1.54
Resolution range (Å)	46.59–1.25 (1.28–1.25)
Space group	C 1 2 1
Unit cell	92.06 Å, 52.24 Å, 64.96 Å 90°, 134.2°, 90°
Total reflections	512 709
Unique reflections	57 570 (2 763)
Multiplicity	8.69
Completeness (%)	93.5 (61.2)
<I/σI > overall	26.1 (3.7) 1.18 (at 1.25 Å)
Mosaicity	0.193
Wilson B‐factor (Å^2^)	9.2
Anisotropy	0.883
R_sym_ ^a^ (%)	4.3 (40.0)

Refinement and quality	
Reflections used in refinement	54 566 (2 626)
Reflections used for R‐free	2 903 (137) (5.04%)
R_work_ ^b^	0.106
R_free_ ^c^	0.132
Number of non‐hydrogen atoms	2 419
macromolecules	2 048
ligands	43
solvent	328
Protein residues	240
RMSD bond length (Å)	0.008
RMSD bond angles (°)	1.28
RMSD chirality	0.079
RMSD planarity	0.007
Fo‐Fc, Fo‐Fc free	0.983, 0.977

Ramachandran plot	
Ramachandran favored (%)	97
Ramachandran allowed (%)	3
Ramachandran outliers (%)	0
Rotamer outliers (%)	1
All atom clashscore	1
RSRZ outliers	2.9%
Average B‐factors (Å^2^)	16.0
macromolecules	12.8
ligands	9.4
solvent	29.4

*Note*: The numbers in parentheses represent statistics in the highest resolution shell.

^a^ *R*_sym_ = ∑|*I*_i_ ‐ < |>|/∑|*I*_i_| where *I*_i_ is the intensity of the *i*th measurement, and < |>is the mean intensity for that reflection.

^b^ *R*_work_ = ∑‖*F*_o_| − |*F*_c_|/∑|*F*_o_|, where *F*_o_ and *F*_c_ are the observed and calculated structure factors for data used for refinement, respectively.

^c^ *R*_free_ = ∑‖*F*_o_| − |*F*_c_|/∑|*F*_o_| for 5% of the data not used at any stage of structural refinement.

**FIGURE 1:**
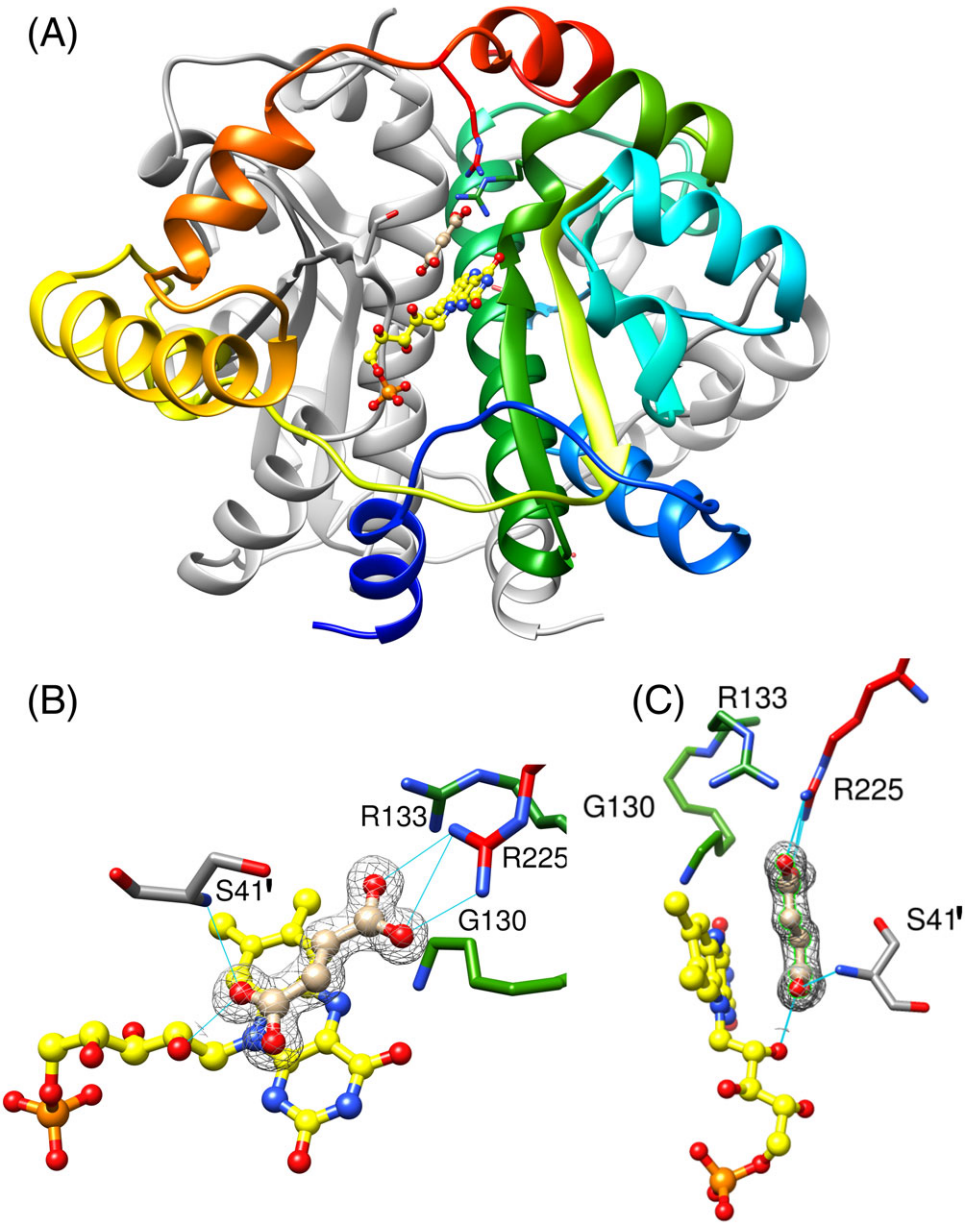
X‐ray structure of fumarate bound to NfsA. (A) Ribbon diagram of NfsA dimer, with bound fumarate. One subunit is in gray and the other is in rainbow colors blue to red from N‐ to C‐ terminus. The FMN cofactor is shown as ball and stick, with C atoms in yellow, N blue, oxygen red, and phosphorus orange. The side chains that interact with the bound fumarate are shown as sticks, with carbon atoms colored as the ribbon backbone, and heteroatoms as for FMN. The ligand is shown in tan, with oxygen atoms in red. (B,C) Two orientations of fumarate bound to NfsA. The FMN and fumarate are shown in ball and stick, colored as in A. The side chains that interact with fumarate are shown as sticks, labeled and colored as in A, with Ser 41, from the opposite subunit to the other interacting residues, given a prime notation. Cyan lines show the hydrogen bonding to the ligand. The mesh shows the electron density within a radius of 2 Å from the fumarate (level 0.53 e) at 1 sigma.

The structure of the protein in the presence of fumarate is fully symmetrical, with only one subunit in the asymmetric unit, and shows the characteristic dimeric, alpha/beta fold of NfsA (Figure 1A). Each monomer has a core domain of four beta strands surrounded by alpha helices, and a two‐helix excursion domain that crosses the dimer interface. The two monomers form extensive contacts, with FMN cofactors lying on either side of the dimer interface, making contacts with both subunits. The fumarate ligands lie in the active sites, parallel to the central aromatic ring of FMN. There is no change in the backbone of the protein from the free structure,^21^, ^22^ with an RMSD of 0.22 Å between the structures. One of the carboxylate groups of fumarate forms salt bridges with the guanidinium group of Arg 225 and is close to Arg 133 and Gly 130. The other carboxylate group forms hydrogen bonds to the O2' hydroxyl of the FMN ribityl group and the backbone amide of Ser 41' (where the prime sign denotes that Ser 41 comes from the other NfsA subunit to the other residues that co‐ordinate with the ligand) (Figure 1B,C). Hydrophobic interactions or π stacking can occur between the FMN and the central atoms of the acid, which are at 3.2 and 3.6 Å to the N5 of the FMN, a suitable distance for a hydrogen transfer reaction.

### 3.2 Steady‐state kinetics with NfsA

**FIGURE 2:**
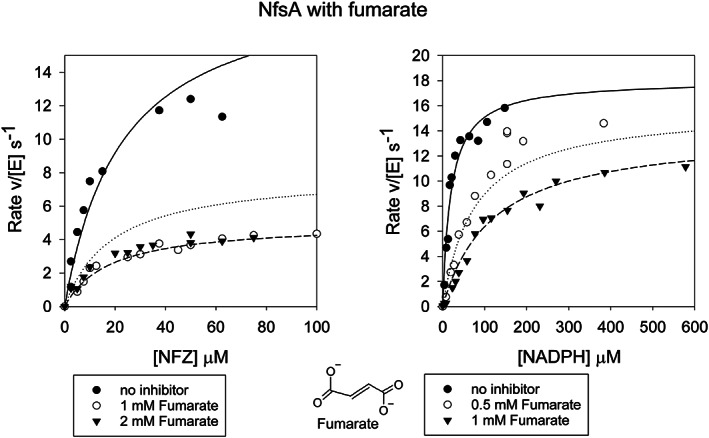
Steady‐state kinetics of NfsA with fumarate. Left: Steady‐state kinetics of NfsA with 100 μM NADPH, varying nitrofurazone, in the presence and absence of fumarate. Right: Steady‐state kinetics of NfsA with nitrofurazone at 75 μM, varying NADPH concentration in the presence or absence of fumarate. The reaction was done in a 10 mM Tris pH 7 buffer, 4.5% DMSO, with total ionic strength 50 mM, at 25°C. The symbols show the data, the lines show the simulated curves to Equation 1, mixed inhibition, with *k*_cat_ 25 s^−1^, *K*_m_ NADPH 29 μM, *K*_m_ NFZ 27 μM, K_i_ NADPH 145 μM, K_i_ NFZ 960 μM.

**FIGURE 3:**
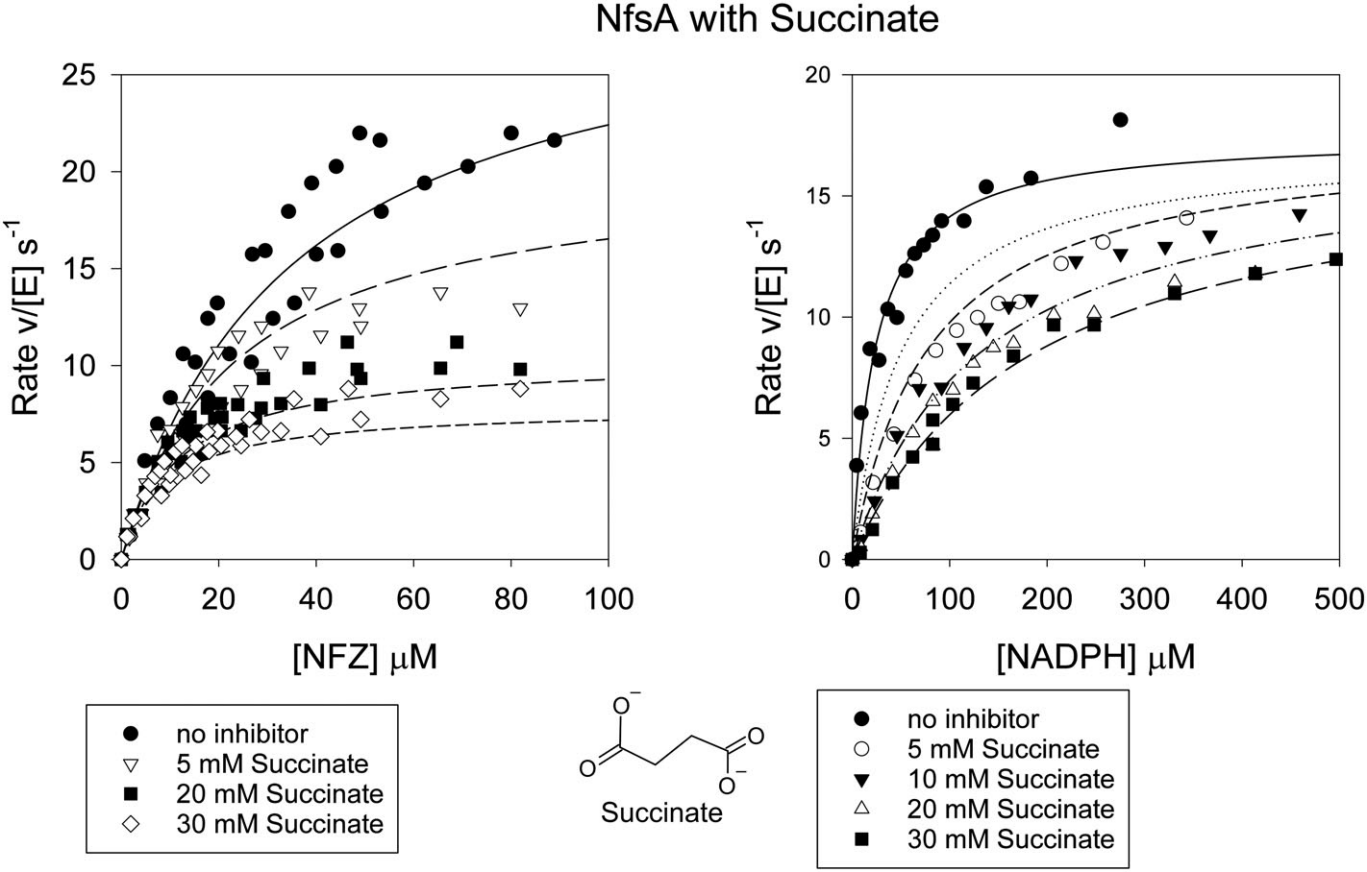
Steady‐state kinetics of NfsA with succinate. Left: Steady‐state kinetics of NfsA with 100 μM NADPH, varying nitrofurazone, in the presence and absence of succinate. Right: Steady state kinetics of NfsA with nitrofurazone at 30 μM, varying NADPH concentration in the presence or absence of succinate. The reactions were done in a 10 mM Tris pH 7 buffer, 4.5% DMSO, with total ionic strength 150 mM, at 25°C. The symbols show the data, the lines show the simulated curves to Equation 1, with *k*_cat_ 51 s^−1^, *K*_m_ NADPH 69 μM, *K*_m_ NFZ 58 μM, *K*_i_ NADPH 4.3 mM.

Steady‐state kinetics studies showed that neither succinate nor fumarate are substrates for NfsA but both act as inhibitors of NfsA, largely competitive with respect to NADPH. In order to ensure that the effects were not due to changes in ionic strength, the reactions were done at the same ionic strength with and without an inhibitor. At 50 mM ionic strength, fumarate gave an inhibition constant of 145 ± 28 μM with respect to NADPH and, six‐fold weaker, 960 ± 390 μM, inhibition with respect to nitrofurazone (Figure 2A,B, Table 2). Succinate showed much weaker inhibition than fumarate, so reactions were repeated with higher concentrations of succinate, up to 30 mM. The reactions with succinate were therefore done at higher total ionic strength, 150 mM. These showed that succinate has a *K*_i_ of 4.3 ± 0.6 mM with respect to NADPH, but no inhibition with respect to nitrofurazone was detected (Figure 3A,B, Table 2). The stronger binding of fumarate compared to succinate is likely to be due to π stacking of the central double bond of fumarate with the FMN ring; all known nitroreductase substrates are aromatic. The weak binding of succinate explains why NfsA does not act as a succinate dehydrogenase.

**TABLE 2:** Steady‐state kinetic data for NfsA with nitrofurazone and NADPH in the presence of fumarate or succinate.

Inhibitor	Ionic strength	*k*_cat_ (s^−1^)	*p*	*K*_m_ (μM)	*p*	*k*_cat_/*K*_m_ (s^−1^ μM^−1^)	*p*	*K*_i_ (μM)	*p*
Fumarate	50	25 ± 2	<.0001	27 ± 6 (NFZ)	<.0001	0.93 ± 0.1	<.0001	960 ± 390 (NFZ)	.017
				29 ± 6 (NADPH)	<.0001	0.86 ± 0.1	<.0001	145 ± 28 (NADPH)	<.0001
Succinate	150	51 ± 6	<.0001	58 ± 10 (NFZ)	<.0001	0.88 ± 0.04	<.0001	‐	
				69 ± 16 (NADPH)	<.001	0.74 ± 0.07	<.0001	4300 ± 600 (NADPH)	<.0001

*Note*: Steady‐state kinetic data for NfsA with nitrofurazone and NADPH in the presence of fumarate or succinate at 10 mM Tris, pH 7.0, 4.5% DMSO at 25°C and the ionic strength shown. A series of kinetic experiments were done at different inhibitor concentrations, either varying NADPH concentration at constant nitrofurazone, or varying nitrofurazone concentration at 100 μM NADPH. The rates of all the reactions were fitted simultaneously to Equation 1, using non‐linear regression in Sigmaplot 14.5, with equal weighting of points, giving the statistics shown.

### 3.3 No effect with NfsB

**FIGURE 4:**
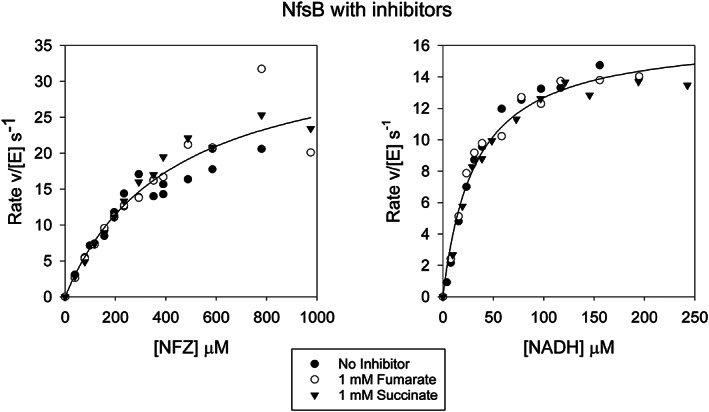
Steady‐state kinetics of NfsB with fumarate and succinate. Left: Steady‐state kinetics of NfsB with NADH at 100 μM, varying the nitrofurazone concentration in the presence or absence of 1 mM succinate or 1 mM fumarate. The line shows the Michaelis–Menten curve with *k*_cat_ 16.7  s^−1^ and *K*_m_ NADH 32 μM. Right: Steady state kinetics of NfsB with 300 μM nitrofurazone , varying the concentration of NADH, in the presence and absence of 1 mM succinate or 1 mM fumarate. The line shows the Michaelis–Menten curve with *k*_cat_ 36 s^−1^ and *K*_m_ NFZ 20 μM. The reactions were done in a 10 mM Tris pH 7 buffer, 4.5% DMSO, with total ionic strength 50 mM, at 25°C. The symbols show the data, black circles – no inhibitor, white circles‐ 1 mM fumarate, inverted triangles‐ 1 mM succinate.

**FIGURE 5:**
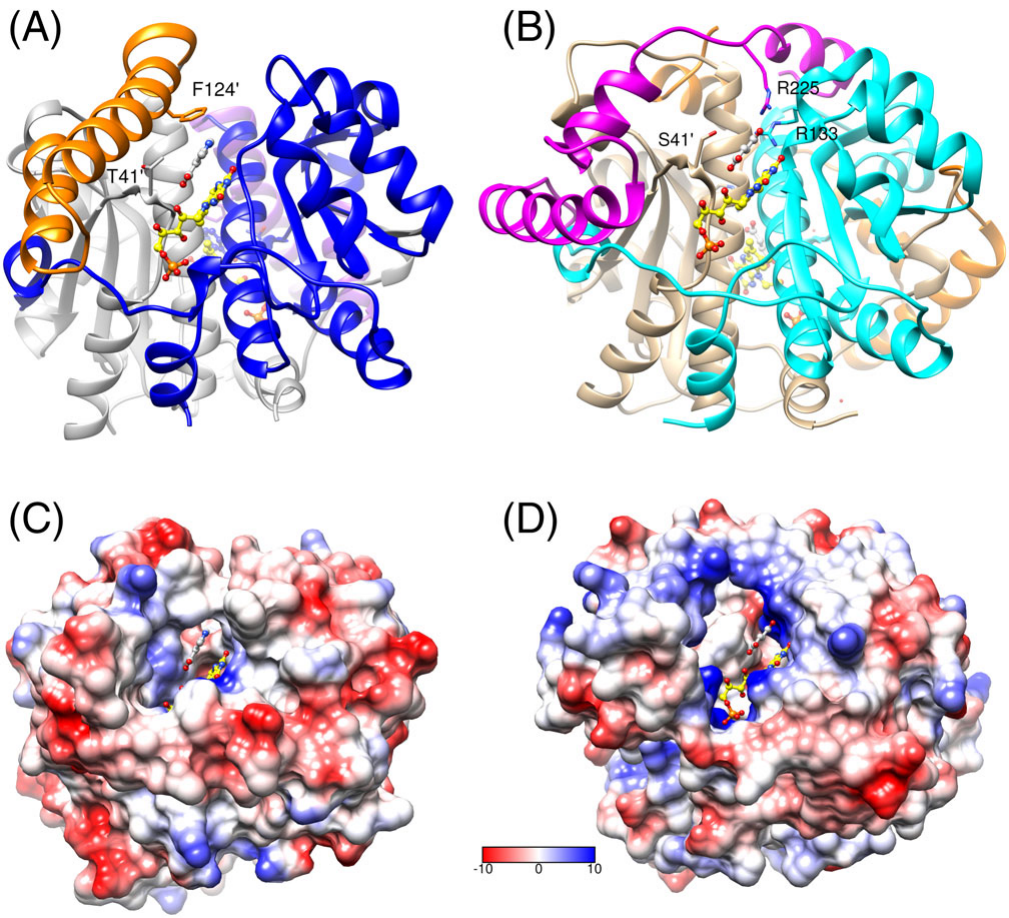
Comparison of the X‐ray structures of NfsB and NfsA. (A) Ribbon diagram of NfsB dimer with bound nicotinate, from 1ICR,^26^ in the same orientation as NfsA in Figure 1. The core residues of the two subunits are in gray and blue, with residues 95–132, not found in NfsA, in in gold and magenta, respectively. The FMN cofactor is shown as ball and stick, with C atoms in yellow, N blue, O red, and P orange. The side chains that interact with the bound nicotinate are labeled and shown as sticks, with carbon atoms colored as the ribbon backbone, and heteroatoms as for FMN. The nicotinate ligand is shown in ball and stick with C atoms in gray and others in CPK colors. (B) Ribbon diagram of NfsA dimer with bound fumarate, in the same orientation as NfsB. One subunit is in tan and the other in cyan, with residues 180–240, not found in NfsB, in gold and magenta, respectively. The side chains that interact with the bound fumarate are labeled and shown as sticks, with carbon atoms colored as the ribbon backbone, and heteroatoms as for FMN. The FMN is colored as in A with the fumarate in ball and stick with C atoms in gray and oxygen in red. (C,D) Coulombic surface representation of NfsB, and NfsA respectively, in the same orientation as in A and B. The FMN and ligands are shown as ball and stick, colored as in A and B. The surface is colored red through white to blue corresponding to negative, through uncharged to positive charge.

Kinetic assays of NfsB showed no effect of either fumarate or succinate at 1 mM concentration (Figure 4, Table S1). Comparison of the structure of NfsB^26^ with that of NfsA (Figure 5) shows that, despite limited sequence homology, they have a similar core structure of ~180 amino acids containing five helices and four beta strands, intertwined in a stable dimer. The FMN cofactor packs on the core of the protein, interacting with both subunits, and has similar interactions in NfsA and NfsB. However, while the known range of substrates for both enzymes are similar and these stack upon the FMN ring in a similar way, they interact differently with the two proteins. NfsB contains a 2‐ helix insertion (residues 95–132, colored gold and magenta for the two different subunits in Figure 5) before the long, central helix of the core structure, with one helix stacking against the side of the substrate. In contrast, NfsA has an approximately 60 aa C‐terminal extension (residues 180–240), after the chain has crossed the dimer interface.^9^ This region forms the phosphate‐binding pocket for the NADPH cofactor and then extends back across the dimer interface, over the substrate, so that a different subunit is over the substrate from that in NfsB. The extensions make the shape of the active site, the channels into it, and the interactions with the substrates very different between the two enzymes.^44^ The active site cavity of NfsB is smaller and much less positively charged than that of NfsA, (Figure 5C,D). In both enzymes the substrates interact with a hydroxyl group at position 41', Ser 41' in NfsA, and Thr 41' in NfsB. In NfsB, substrates interact with Phe 124' in the same subunit as Thr 41'^26^, ^27^ whereas in NfsA substrates interact with Arg 225 of the other subunit,^22^, ^23^ neither of which has a counterpart in the other enzyme (Figure 5A,B). The absence of the charged residues Arg 225 and Arg 133 in NfsB, means that it does not bind fumarate.

### 3.4 In vivo effect of fumarate

While the dissociation constant for fumarate from NfsA is high, as shown in the *K*i measurements above, it is within the cellular concentration in *E. coli* (~0.1 mM *cf* succinate 0.5 mM)^45^ and so it would be a regulator *in vivo*. One possible role for this may be in anaerobic respiration, when fermentation is not used and fumarate acts as the terminal electron acceptor. Under these conditions, NADH dehydrogenase I reduces menaquinone to menaquinol using NADH, translocating 4 protons in this reaction, so allowing ATP generation. Fumarate reductase reoxidises the menaquinol so the process can continue. Inhibition of the reduction of quinones by NfsA would prevent a futile cycle, without proton translocation. In the longer term, *nfs*A gene expression is repressed in the stationary phase by a small RNA, *sdsN* ^46^ as is the upstream gene in the operon, *ybjC*, which is thought to be a membrane‐bound oxidase. NfsB, which also reduces quinones, has a higher *K*_m_ for most substrates (for example K_m_ NFZ of NfsB in Table S1 *cf* K_m_ NFZ of NfsA in Table 2) and so repression of this enzyme may be less important.

The structural extensions of *E. coli* NfsA and NfsB are characteristic of the two large subfamilies of nitroreductase proteins. Arg 225 is conserved in 61% of the NfsA subgroup,^9^ thus fumarate repression is likely to be observed in most of the NfsA subfamily of proteins. The presence and differences in concentrations of such inhibitors, as well as of intrinsic substrates, have been suggested to explain some of the differences observed in the relative efficacy of cell killing with mutant enzymes in bacteria compared to that in eukaryotic cells.^10^, ^47^ The identification of natural inhibitors of NfsA and NfsB *in vivo* is thus important in designing enzymes for use with prodrugs or theranostic reagents and for biosynthetic or detoxifying reactions as well as for elucidating the possible roles of these enzymes in their host bacteria.

## AUTHOR CONTRIBUTIONS

Martin A. Day, and David Jarrom, supervised by Eva I. Hyde and Peter F. Searle, did kinetics experiments. Crystallography was done by Martin A. Day and David Jarrom, supervised by Scott A. White. The paper was written by Eva I. Hyde.

## CONFLICT OF INTEREST

No commercial interest.

### PEER REVIEW

The peer review history for this article is available at https://publons.com/publon/10.1002/prot.26451.

## Supporting information


**Supplementary Table S1:** Steady‐state kinetic data for NfsB with nitrofurazone and NADH. A series of kinetic experiments were done with and without 1 mM succinate or fumarate, either varying NADH concentration at 300 μM  nitrofurazone, or varying nitrofurazone concentration at 100 μM NADPH, in 10 mM Tris, pH 7.0, 50 mM ionic strength, 4.5% DMSO at 25°C. The rates of the reactions with each substrate were fitted to the Michaelis Menten equations for different types of inhibition, using non‐linear regression in Sigmaplot 14.5, with equal weighting of points. No inhibition was seen so all rates were fitted to the equation without inhibition, giving the statistics shown.

## Data Availability

The crystallographic data has been deposited in the Protein Data Bank with accession code 8AJX.
