# Supplementary material for: Oxygen‐insensitive nitroreductase E. coli NfsA, but not NfsB, is inhibited by fumarate
Source: Proteins. 2022 Dec 13;91(5):585–92. doi: 10.1002/prot.26451 (PMC10953011; doi:10.1002/prot.26451)
Supplement: Supplementary file 1 — Supplementary Table S1: Steady‐state kinetic data for NfsB with nitrofurazone and NADH. A series of kinetic experiments were done with and without 1 mM succinate or fumarate, either varying NADH concentration at 300 μM nitrofurazone, or varying nitrofurazone concentration at 100 μM NADPH, in 10 mM Tris, pH 7.0, 50 mM ionic strength, 4.5% DMSO at 25°C. The rates of the reactions with each substrate were fitted to the Michaelis Menten equations for different types of inhibition, using non‐linear regression in Sigmaplot 14.5, with equal weighting of points. No inhibition was seen so all rates were fitted to the equation without inhibition, giving the statistics shown. [file PROT-91-585-s001.docx]

| Substrate | k_cat_ app  (s^-1^) | P | K_m_ app  (µM) | P | k_cat_/K_m_  (s^-1^ µM^-1^) | P |
| --- | --- | --- | --- | --- | --- | --- |
| NFZ | 36 ± 2 | < 0.0001 | 420 ± 60 | < 0.0001 | 0.084 ± 0.006 | <0.0001 |
| NADH | 16.7 ± 0.4 | < 0.0001 | 32 ± 2 | < 0.0001 | 0.53 ± 0.03 | < 0.0001 |

Supplementary Table S1: Steady-state kinetics parameters for NfsB with NADH and nitrofurazone. A series of kinetic experiments were done at with and without 1 mM succinate or fumarate, either varying NADH concentration at 300 µM nitrofurazone, or varying nitrofurazone concentration at 100 µM NADPH, at 10 mM Tris, pH 7.0, 50 mM ionic strength, 4.5% DMSO at 25°C. The rates of the reactions with each substrate were fitted to the Michaelis Menten equations for different types of inhibition, using non-linear regression in Sigmaplot 14.5, with equal weighting of points. No inhibition was seen so all rates were fitted to the equation without inhibition, giving the statistics shown.
